# The diagnostic value of prostate health index combined with soluble e-cadherin for prostate cancer

**DOI:** 10.3389/fendo.2025.1531866

**Published:** 2025-09-30

**Authors:** Yeasin Ahamed, Marofe Hossain, Shantanu Baral, Jing Chen, Weigui Sun

**Affiliations:** ^1^ Department of Urology Surgery, The Affiliated Hospital of Yangzhou University, Yangzhou, Jiangsu, China; ^2^ Clinical Medical College, Yangzhou University, Yangzhou, Jiangsu, China

**Keywords:** PHI (prostate health index), sE-cadherin (soluble e-cadherin), prostate cancer (pca), diagnostic biomarker, metastasis

## Abstract

**Objective:**

To assess the diagnostic value of combining prostate health index (PHI) and soluble epithelial cadherin (sE-cadherin) in prostate cancer (PCa) detection.

**Methods:**

This study included 250 benign prostatic hyperplasia (BPH) and 250 PCa patients (2020–2024). PCa patients were categorized by disease stage (I-II:115; III-IV:135), bone metastasis (non-metastatic:171; metastatic:79), and Gleason score (≤8:136; >8:114). Serum sE-cadherin (ELISA), tPSA, fPSA, and p2PSA (chemiluminescence) were measured; PHI was calculated. ROC curves evaluated diagnostic performance.

**Results:**

sE-cadherin, tPSA, p2PSA, and PHI levels were significantly higher in PCa vs. BPH (P<0.05), with further elevations in advanced stages, metastatic cases, and Gleason >8 (P<0.05). ROC analysis demonstrated AUCs of 0.719 (sE-cadherin), 0.761 (PHI), and 0.792 (combined), indicating superior diagnostic accuracy for the combination.

**Conclusion:**

Combining sE-cadherin and PHI enhances PCa detection accuracy, correlating with disease severity, metastasis, and aggressiveness.

## Introduction

Prostate cancer (PCa) is a significant global health concern, being the second most common malignancy among men and a leading cause of cancer-related mortality. The disease’s slow progression necessitates early detection and effective risk stratification to improve outcomes. Recent advancements in diagnostic methods and screening practices are crucial for managing this prevalent condition. PCa incidence is highest in regions with aging populations, particularly in North America and Europe, with approximately 1.41 million new cases diagnosed in 2020 (1). Key risk factors include advanced age, family history, and genetic predispositions, with lifestyle factors such as diet and physical activity also playing a role (2). Current diagnostic strategies involve PSA testing, Gleason scoring, and advanced imaging techniques like MRI and PSMA-PET, which enhance the identification of significant tumors (3). The integration of biomarkers into screening protocols is expected to reduce overdiagnosis and improve patient management.Current clinical diagnostic pathways typically begin with serum prostate-specific antigen (PSA) testing and digital rectal examination (DRE) for initial screening. In cases with elevated PSA or suspicious DRE findings, multiparametric magnetic resonance imaging (mpMRI) is increasingly used to identify suspicious lesions and guide subsequent targeted biopsies, which remain the diagnostic gold standard.While this strategy has improved detection, the reliance on PSA testing alone is fraught with limitations, including low specificity and leading to overdiagnosis and overtreatment of indolent tumors.This underscores the critical need for more specific biomarkers to be integrated into screening protocols to improve risk stratification and patient management (4, 5).

The occurrence and progression of prostate cancer (PCa) are influenced by a multifaceted interplay of factors, including genetic predispositions, age, and family history. Age is a primary risk factor, with the majority of diagnoses occurring in men over 65 years, highlighting the significance of age-related changes in disease development (6, 7). As men age, they are more likely to accumulate genetic mutations in prostate cells, which can lead to cancer. Additionally, family history is a crucial determinant, as men with first-degree relatives diagnosed with PCa face a significantly increased risk. Genetic mutations, particularly in the BRCA1 and BRCA2 genes, have been linked to a higher likelihood of developing aggressive forms of prostate cancer, underscoring the role of hereditary factors in the disease’s progression (8).Furthermore, lifestyle choices, diet, and environmental exposures, including infections, may also contribute to the risk of developing PCa, although these factors require further investigation to fully understand their impact (6, 9). Overall, the complex interplay of these elements necessitates a comprehensive approach to understanding and managing prostate cancer risk.

Prostate cancer (PCa) poses significant challenges for early diagnosis due to its often-asymptomatic early stages and the complexity of its metastatic behavior.Late-stage diagnosis, particularly when metastasis occurs, severely impacts treatment options and survival rates.Many patients are diagnosed only after metastasis, often to bones, which complicates treatment and worsens prognosis (10).Current screening methods, primarily serum prostate-specific antigen (PSA) testing, have limitations and do not consistently lead to early detection (11). Emerging technologies, such as electrochemical biosensors and nanotechnology, show promise for earlier and more accurate detection of Pca (12, 13). Risk stratification tools and next-generation sequencing are being developed to personalize treatment and improve early diagnosis (14). Despite advancements, there remains a critical need for universal, non-invasive diagnostic tools that can effectively identify PCa in its early stages (15).While significant progress has been made in understanding and diagnosing prostate cancer, the inherent challenges of tumor growth dynamics and the timing of metastasis continue to hinder early detection efforts, necessitating ongoing research and innovation in screening methodologies.

The role of cell adhesion molecules, particularly E-cadherin, is crucial in the initiation and progression of prostate cancer (PCa).E-cadherin maintains epithelial integrity, and its loss is associated with tumor progression.This response synthesizes findings from recent studies to elucidate the biological mechanisms involved.E-cadherin is essential for strong intercellular adhesion, which regulates cellular proliferation and maintains tissue architecture (16).Loss of E-cadherin expression is linked to epithelial-mesenchymal transition (EMT), a process that enhances cancer cell invasiveness and metastasis (17). Epigenetic alterations, including DNA methylation and histone modifications, contribute to the downregulation of E-cadherin in Pca (18). N-cadherin, an alternative cadherin, is often upregulated in advanced PCa, promoting tumor progression through epigenetic reprogramming (19).Chronic inflammation modifies the tumor microenvironment, further facilitating EMT and disrupting cell adhesion mechanisms (20). While E-cadherin loss is a hallmark of aggressive PCa, some studies suggest that targeting alternative pathways, such as N-cadherin, may offer therapeutic avenues to counteract the effects of E-cadherin loss and improve patient outcomes (17).

The downregulation of E-cadherin is a critical event in the epithelial-mesenchymal transition (EMT), significantly contributing to cancer metastasis.This transition allows epithelial cells to lose their adhesive properties, facilitating tumor cell detachment and invasion into surrounding tissues.E-cadherin loss is associated with increased tumor aggressiveness and poor differentiation, as evidenced by studies showing that high-grade tumors exhibit weak E-cadherin expression (21). In oral squamous cell carcinoma, a significant correlation was found between E-cadherin loss and poor survival outcomes, highlighting its prognostic value (22).In oral squamous cell carcinoma, a significant correlation was found between E-cadherin loss and poor survival outcomes, highlighting its prognostic value (22, 23).Additionally, E-cadherin downregulation activates various transcription factors, such as Twist, which further drive the metastatic process (24). While the loss of E-cadherin is a hallmark of EMT and metastasis, some studies suggest that not all tumors with reduced E-cadherin expression will exhibit aggressive behavior, indicating a complex interplay of factors influencing cancer progression.

Elevated levels of soluble E-cadherin (sE-cadherin) in the bloodstream have emerged as a significant biomarker for tumor progression, particularly in prostate cancer (PCa). Unlike its membrane-bound form, sE-cadherin promotes the dissociation of cell-cell junctions, facilitating tumor invasion and metastasis. Increased sE-cadherin levels correlate with advanced disease stages and poor prognosis in various cancers, including Pca (25).Critically, this theoretical potential is supported by clinical evidence.Studies have specifically demonstrated that serum levels of sE-cadherin are significantly elevated in patients with prostate cancer compared to those with benign conditions, and higher levels are correlated with advanced disease stage, metastasis, and poorer prognosis (23, 26, 27).This existing body of research establishes sE-cadherin as a promising circulating biomarker worthy of further investigation in combination with other advanced diagnostic tools.

The downregulation of E-cadherin is linked to epithelial-to-mesenchymal transition (EMT), a process associated with enhanced migratory and invasive properties of tumor cells (28, 29). Measuring circulating sE-cadherin could serve as a non-invasive indicator of tumor aggressiveness, aiding in early detection and monitoring of PCa progression (25).Studies indicate a negative association between E-cadherin expression and tumor grade, suggesting its potential utility in assessing cancer severity (30).While sE-cadherin shows promise as a biomarker, its role in promoting tumor progression raises concerns about therapeutic strategies targeting E-cadherin pathways, necessitating further research to balance its dual roles in cancer biology.

Prostate-specific antigen (PSA) testing has significantly impacted prostate cancer detection since its introduction, yet it is fraught with limitations, particularly regarding specificity. Elevated PSA levels can arise from benign conditions, leading to unnecessary interventions and patient distress. Elevated PSA can result from benign prostatic hyperplasia (BPH) or prostatitis, contributing to high false-positive rates.The reliance on PSA has led to overdiagnosis and overtreatment of low-grade cancers, raising concerns about the associated adverse effects (31). New biomarkers, such as the Prostate Health Index and 4Kscore, are being developed to enhance specificity and reduce unnecessary biopsies (31).These biomarkers aim to provide a more personalized approach to screening, potentially improving patient outcomes (32).While PSA remains a cornerstone in prostate cancer screening, the need for more specific biomarkers is critical to mitigate the risks of overdiagnosis and overtreatment.

The Prostate Health Index (PHI) enhances prostate cancer detection by integrating total PSA, free PSA, and (-2) proPSA, significantly improving diagnostic accuracy, especially in the “gray zone” of PSA levels (4–10 ng/mL). This refined tool reduces unnecessary biopsies and better discriminates between benign and malignant conditions.PHI has shown a sensitivity of 82% and specificity of 84% in detecting prostate cancer, with an optimal cutoff of 43 points (33, 34).In a large cohort study, higher PHI scores (≥35) correlated with a 23% cancer detection rate, compared to 7.9% in lower scores (26). PHI aids in shared decision-making, with 83% of patients opting against biopsy when PHI indicated lower risk (26). Combining PHI with multiparametric MRI further enhances diagnostic performance, reducing unnecessary procedures by approximately 20% (35). While PHI represents a significant advancement in prostate cancer diagnostics, some studies suggest that combining it with other imaging techniques may yield even better outcomes, indicating a potential area for further research and clinical application.

The combination of sE-cadherin and PHI as biomarkers in prostate cancer (PCa) presents a promising approach for enhancing early detection and risk stratification. While PHI aids in assessing the likelihood and aggressiveness of PCa, sE-cadherin provides critical insights into the tumor’s metastatic potential. sE-cadherin has been shown to significantly influence the metastatic behavior of PCa cells, promoting cell detachment and enhancing migration and invasion capabilities (36).sE-cadherin has been shown to significantly influence the metastatic behavior of PCa cells, promoting cell detachment and enhancing migration and invasion capabilities (37). The Prostate Health Index (PHI) integrates total PSA, free PSA, and (-2) proPSA levels, offering a more nuanced risk assessment for PCa presence and aggressiveness (38). Studies indicate that PHI can effectively differentiate between indolent and aggressive tumors, aiding in treatment decision-making (39). Combining these biomarkers could lead to more personalized treatment strategies, allowing clinicians to identify patients at higher risk for aggressive disease and metastasis. However, the integration of these biomarkers into clinical practice requires further validation and standardization. This study investigates the diagnostic efficacy of combining PHI with sE-cadherin for PCa, with the aim of providing new reference criteria for the early diagnosis of PCa.

## Materials and methods

### General data

**Figure 1:**
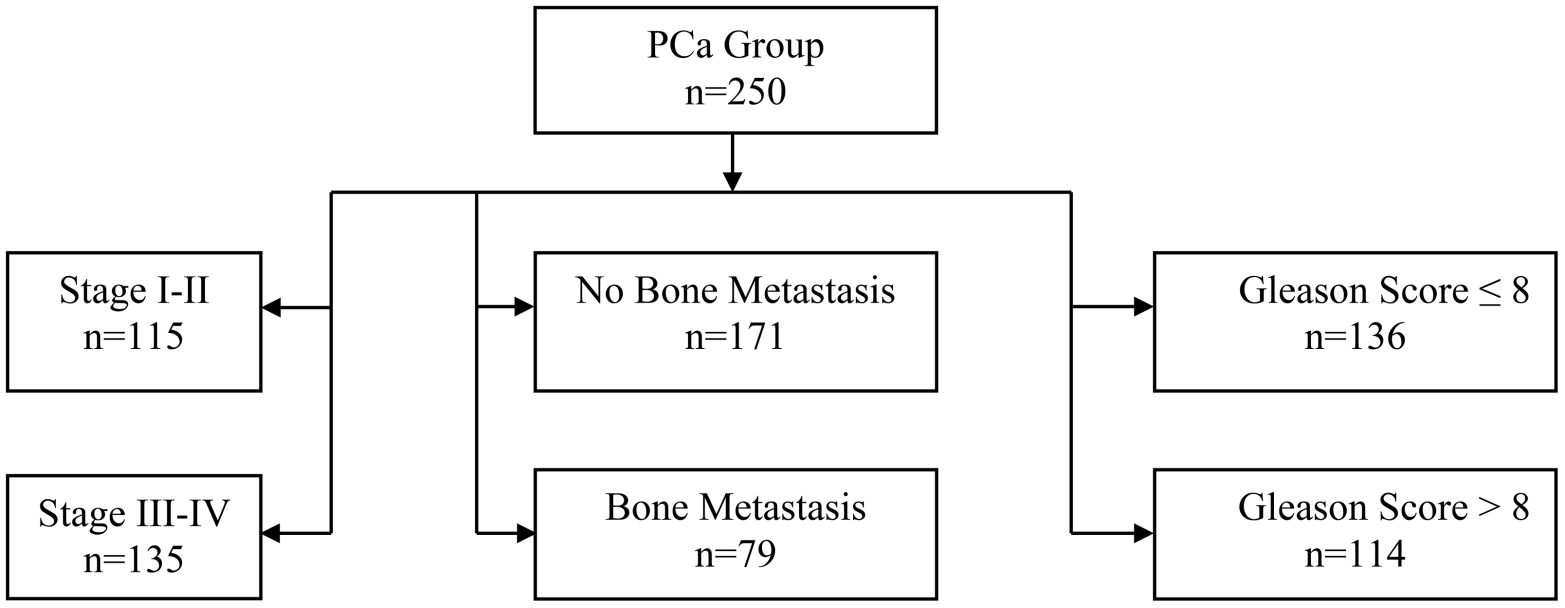
Flowchart of 250 prostate cancer patients. Groups were divided by bone metastasis (No: n=171, Yes: n=79). No metastasis patients were staged (I-II: n=115, III-IV: n=135). Gleason scores were stratified as ≤8 (n=136) and >8 (n=114).

This study included a total of 500 patients who were admitted to the Affiliated Hospital of Yangzhou university between January 2020 to September 2024.Among them, 250 patients diagnosed with benign prostatic hyperplasia (BPH) were selected as the BPH group, and 250 patients diagnosed with prostate cancer (PCa) were selected as the PCa group. The PCa group was further divided into subgroups based on cancer stage, bone metastasis, and Gleason score (As shown in **Figure 1**).

### Inclusion criteria

① Met the diagnostic criteria for prostate cancer according to the “Prostate Cancer Clinical Guidelines” by the European Association of Urology (EAU) or the American Urological Association (AUA).② Patients diagnosed with BPH or PCa based on histopathological examination. Availability of complete and comprehensive medical records.④ All participants provided informed consent, agreed, and voluntarily participated in the study.

### Exclusion criteria

① Presence of hypertension, diabetes, or other significant comorbidities. History of taking anti-cancer drugs, anti-androgens, 5α-reductase inhibitors, or undergoing chemical castration within three months prior to inclusion.③ Patients with known coagulation disorders. History of prostate surgery. Presence of concurrent urinary system infections.⑥ Patients who withdrew from the study midway.⑦ Patients who had undergone cystoscopy, catheterization, or similar examinations within one week prior to inclusion. Patients with a history of other types of malignant tumors.⑨ Patients with Parkinson’s disease or dementia.

### Instruments and reagents

Total prostate-specific antigen (tPSA), free prostate-specific antigen (fPSA), and prostate-specific antigen isoform 2 (p2PSA): These were analyzed using a chemiluminescence analyzer (manufactured by Wuhan Fine Biotech Co., Ltd.).

Soluble epithelial cadherin (sE-cadherin): The sE-cadherin levels were measured using an enzyme-linked immunosorbent assay (ELISA) kit following the manufacturer’s instructions.

### Detection method

For all patients undergoing prostate cancer screening, 3–5 ml of venous blood was collected via venipuncture into standard serum separator tubes (SST) prior to performing digital rectal examination (DRE) or any transurethral instrumentation.To ensure complete clotting, the samples were allowed to stand at room temperature for 30 minutes.The blood samples were then centrifuged at 2,800 r/min (approximately 1,500 RCF) with a centrifuge radius of 10 cm for 10 minutes to separate the serum.The serum aliquots were subsequently stored at -80 °C until analysis for p2PSA, fPSA, tPSA, and sE-cadherin.This standardized protocol was implemented to minimize pre-analytical variability and ensure biomarker stability.

Calculation of Prostate Health Index (PHI): The PHI was calculated using the following formula: PHI= (p2PSA/fPSA) × √tPSA.

### Statistical analysis

Continuous variables such as sE-cadherin, tPSA, fPSA, p2PSA, and PHI were expressed as mean ± standard deviation (SD).Differences between the BPH and PCa groups and their subgroups were analyzed using the student’s t-test or ANOVA.The diagnostic performance of sE-cadherin, PHI, and their combination for PCa was evaluated using receiver operating characteristic (ROC) curves.The area under the curve (AUC) was used to assess diagnostic accuracy, and comparisons were made using the Delong test.A P-value <0.05 was considered statistically significant. Statistical analysis was performed using SPSS 26.0 software.

## Results

### Comparison of sE-cadherin, tPSA, fPSA, p2PSA levels, and PHI between BPH and PCa groups

The PCa group shows significantly higher levels of sE-cadherin, tPSA, p2PSA, and PHI compared to the BPH group, which suggests that these biomarkers are elevated in prostate cancer patients.The fPSA levels, although slightly higher in the PCa group, show a smaller difference compared to the other markers.Overall, the biomarkers, especially PHI, provide a strong differentiation between BPH and prostate cancer patients, with all p-values being highly significant (P < 0.05). as shown in **Table 1**.

**Table 1:** Comparison of sE-cadherin, tPSA, fPSA, p2PSA levels, and PHI between BPH and PCa patients.

Group	N	sE-cadherin/(μg/L)	tPSA/(ng/mL)	fPSA/(ng/mL)	p2PSA/(ng/mL)	PHI
BPH	250	6.237 ± 1.001	6.20 ± 1.12	0.937 ± 0.154	13.74 ± 2.41	36.85 ± 5.16
PCa	250	12.44 ± 2.47	9.84 ± 1.01	0.962 ± 0.110	22.84 ± 4.57	75.35 ± 8.49
t value		36.86	38.20	2.09	27.87	61.27
P value		<0.001	<0.001	0.037	<0.001	<0.001

### Comparison of sE-cadherin, tPSA, fPSA, p2PSA Levels, and PHI in PCa Patients with Different Stages

sE-cadherin, tPSA, p2PSA, and PHI levels are significantly higher in patients with advanced tumor stages (III-IV) compared to those in early stages (I-II) (P<0.05). However, the difference in fPSA levels between the two groups was not statistically significant (P>0.05), as shown in **Table 2**.

**Table 2:** Comparison of sE-cadherin, tPSA, fPSA, p2PSA levels, and PHI in patients with prostate cancer at different stages.

Tumor stage	N	sE-cadherin/(μg/L)	PSA/(ng/mL)	fPSA/(ng/mL)	p2PSA/(ng/mL)	PHI
I~II	115	11.35 ± 0.93	9.05 ± 0.54	0.95 ± 0.106	20.89 ± 0.53	67.73 ± 6.14
III~IV	135	13.15 ± 1.26	10.15 ± 0.51	0.95 ± 0.095	23.65 ± 1.77	79.78 ± 1.88
t value		12.62	16.69	0.098	16.12	21.62
P value		<0.001	<0.001	0.921	<0.001	<0.001

### Comparison of sE-cadherin, tPSA, fPSA, p2PSA levels, and PHI in PCa patients with and without bone metastasis

Patients with bone metastasis have significantly higher levels of sE-cadherin, tPSA, p2PSA, and PHI compared to those without bone metastasis. The difference in fPSA levels between the two groups, while statistically significant, is smaller compared to the other biomarkers. All reported P-values are <0.05 **(as shown in** **Table 3**), indicating significant differences in the biomarker levels between the two groups. This suggests that patients with bone metastasis tend to exhibit higher levels of these markers.

**Table 3:** Comparison of sE-cadherin, tPSA, fPSA, p2PSA levels, and PHI between prostate cancer patients with and without bone metastasis.

Metastasis	N	sE-cadherin/(μg/L)	tPSA/(ng/mL)	fPSA/(ng/mL)	p2PSA/(ng/mL)	PHI
Non-bone metastasis	171	11.73 ± 1.34	9.57 ± 0.51	0.95 ± 0.10	21.94 ± 1.62	71.76 ± 2.88
Bone metastasis	79	13.99 ± 0.56	10.40 ± 0.33	0.99 ± 0.08	23.74 ± 2.46	80.25 ± 1.21
t value		18.72	13.19	2.46	121.24	25.21
P value		<0.001	<0.001	0.014	<0.001	<0.001

### Comparison of sE-cadherin, tPSA, fPSA, p2PSA levels, and PHI in PCa patients with different Gleason scores

Compared with PCa patients with a Gleason score ≤8, patients with a Gleason score >8 had significantly higher levels of sE-cadherin, tPSA, p2PSA, and PHI (P<0.05). However, the difference in fPSA levels between the two groups was not statistically significant (P>0.05), as shown in **Table 4**.

**Table 4:** Comparison of sE-cadherin, tPSA, fPSA, p2PSA levels, and PHI in prostate cancer patients with different Gleason scores.

Gleason Score	N	sE-cadherin/(μg/L)	tPSA/(ng/mL)	fPSA/(ng/mL)	p2PSA/(ng/mL)	PHI
≤8 Score	136	11.16 ± 0.76	9.33 ± 0.69	0.94 ± 0.09	21.60 ± 1.0	71.63 ± 4.25
>8 Score	114	14.03 ± 0.67	10.32 ± 0.37	0.95 ± 0.095	21.68 ± 0.96	77.13 ± 5.34
t value		31.47	13.77	0.92	12.12	9.06
P value		<0.001	<0.001	0.36	<0.001	<0.001

### ROC curve analysis of the diagnostic value of sE-cadherin combined with PHI for Pca

**Figure 2:**
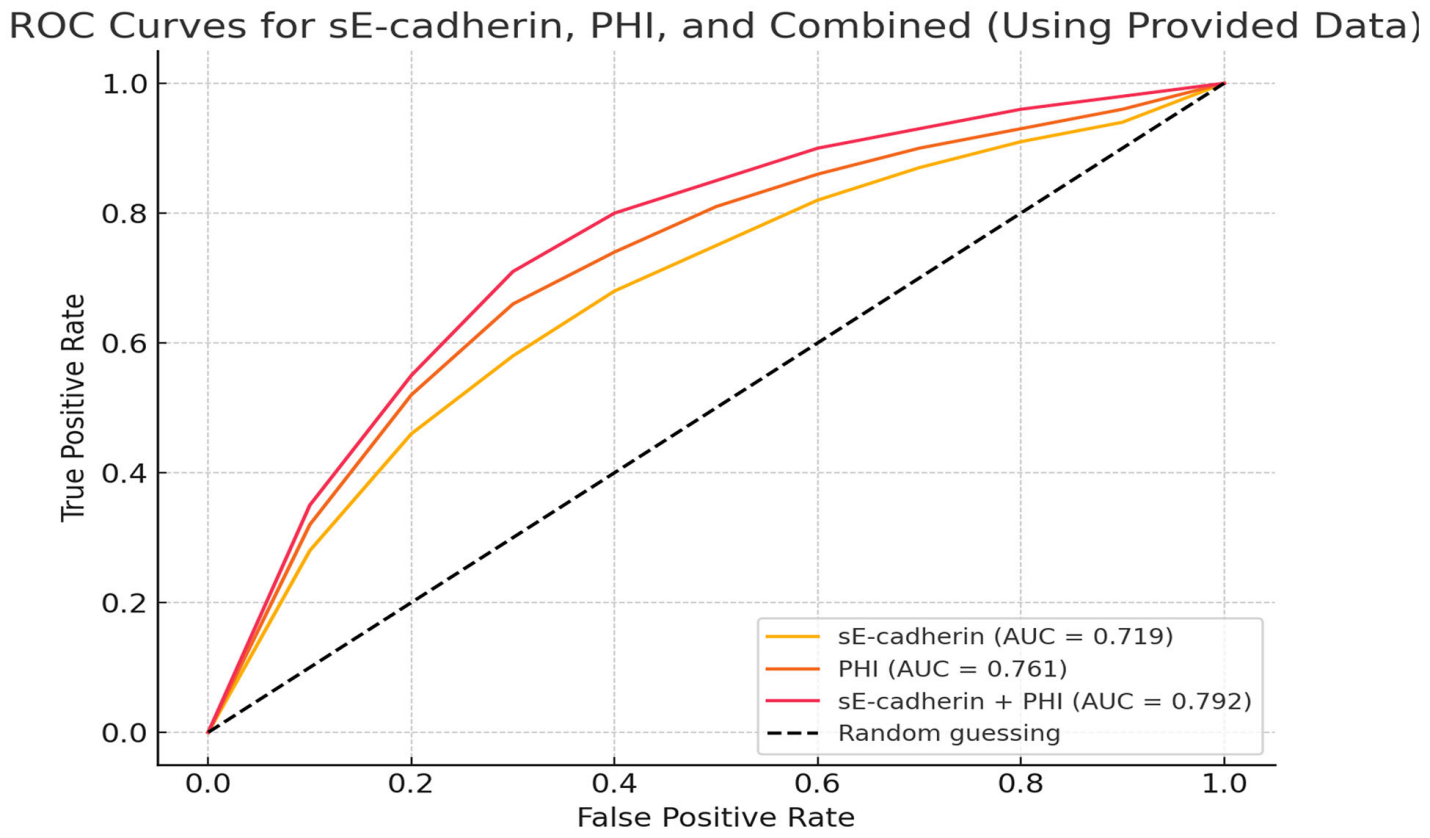
Receiver Operating Characteristic (ROC) curves comparing the diagnostic performance of sE-cadherin, PHI, and the combination of sE-cadherin + PHI.

The area under the ROC curve (AUC) for diagnosing PCa using sE-cadherin, PHI alone, and their combination were 0.719, 0.761, and 0.792, respectively. The AUC for the combined diagnosis was the highest, indicating superior diagnostic performance (**Table 5**, **Figure 2** **).**

**Table 5:** ROC curve analysis of the diagnostic value of combined sE-cadherin and PHI for prostate cancer.

Index	AUC	95%CI	Sensitivity/%	Specificity/%	Youden index	standard error	P value
sE-cadherin	0.719	0.642~0.790	69.8	79.22	0.48	0.12	0.001
PHI	0.761	0.686~0.835	75.23	83.6	0.588	0.09	0.001
sE-cadherin+PHI	0.792	0.712~0.872	94.02	82.3	0.633	0.117	0.001

## Discussion

Prostate cancer (PCa) diagnosis relies heavily on identifying sensitive biomarkers, with the Prostate Health Index (PHI) and soluble epithelial cadherin (sE-cadherin) emerging as significant tools (34, 36). The combination of Prostate Health Index (PHI) and soluble E-cadherin (sE-cadherin) as a diagnostic tool for prostate cancer (PCa) represents a significant advancement in the ongoing effort to enhance diagnostic accuracy (37). By integrating markers that assess both tumor presence (PHI) and metastatic potential (sE-cadherin), This study highlights the diagnostic potential of combining the Prostate Health Index (PHI) with soluble E-cadherin (sE-cadherin) to enhance prostate cancer (PCa) detection and provide insights into disease progression.The combination of these biomarkers significantly improved diagnostic accuracy compared to using either marker alone, as demonstrated by the area under the curve (AUC) in receiver operating characteristic (ROC) analysis.The combined AUC of 0.792 outperformed sE-cadherin (AUC 0.719) and PHI (AUC 0.761), emphasizing the benefit of integrating multiple biomarkers for a more comprehensive assessment of PCa risk.

E-cadherin, located on chromosome 16q22, has a molecular weight of 120 kDa and plays a key role in inhibiting tumor invasion and metastasis.It exists in two forms: tissue-bound and soluble. Tissue-bound E-cadherin can degrade under certain conditions to form sE-cadherin, which is closely associated with tumorigenesis and progression (40, 41). The expression of soluble E-cadherin (sE-cadherin) has been linked to tumor progression in various cancers, including colorectal and gastric cancers, and may also play a significant role in prostate cancer (PCa). Research indicates that higher levels of sE-cadherin correlate with increased tumor size and metastasis, suggesting its potential as a biomarker for cancer aggressiveness (37, 42, 43). Zhu et al. found that sE-cadherin expression in colorectal cancer patients was positively correlated with tumor size and degree of spread (44). In gastric cancer, Zhao et al. demonstrated that lower sE-cadherin levels were associated with poorer survival outcomes, indicating its role in tumor progression (43). The study highlighted that sE-cadherin levels in PCa patients were significantly higher than in benign prostatic hyperplasia (BPH) cases, suggesting its involvement in PCa progression (37, 45). While the findings support the role of sE-cadherin as a potential biomarker for cancer progression, it is essential to consider that not all studies agree on its prognostic value, indicating a need for further research to clarify its role across different cancer types.Elevated soluble E-cadherin (sE-cadherin) levels have been implicated in promoting tumor cell invasion and metastasis, particularly in prostate cancer (PCa).

The elevated levels of sE-cadherin and PHI observed in prostate cancer patients, particularly those with advanced disease, underline the importance of utilizing a multifaceted approach to PCa detection.In this study, the combination of sE-cadherin and PHI yielded an AUC of 0.792, outperforming either marker alone, which indicates that the combination offers a higher sensitivity and specificity for diagnosing prostate cancer.This finding resonates with the growing recognition in the literature that relying on a single biomarker, such as PSA, may not provide sufficient diagnostic accuracy (46).

The Prostate Health Index (PHI) integrates multiple PSA subtypes total PSA, free PSA, and (-2) proPSA into a single index, which has been shown to significantly reduce false positives compared to traditional PSA testing.This reduction in false positives is critical, as overdiagnosis remains a major challenge in prostate cancer screening, often leading to unnecessary biopsies and treatments for indolent tumors that may not have clinical significance (47). PHI, as shown in other studies, has the ability to more accurately discriminate between benign prostatic hyperplasia (BPH) and PCa, especially within the “gray zone” of PSA levels between 4 and 10 ng/mL​ (48, 49). In combination with sE-cadherin, which reflects metastatic potential, the combined test has the potential to identify more aggressive forms of the disease earlier, allowing for more timely and appropriate intervention.A particularly promising application of this biomarker combination is in the diagnostic “gray zone” (PSA 4–10 ng/mL), where the limitations of PSA are most pronounced and the clinical decision for or against a biopsy is most challenging (44). Our findings suggest that the sE-cadherin+PHI model could provide superior discrimination within this range, potentially reducing the rate of unnecessary biopsies for benign conditions. While PHI alone has demonstrated a significant improvement in specificity over PSA (29, 31), the addition of a biologically distinct marker like sE-cadherin, which is directly involved in tumor progression and metastasis, may further refine risk assessment.Future studies focusing specifically on patients within the PSA gray zone are warranted to validate the cutoff values and clinical utility of this combined model for preventing unnecessary procedures while ensuring aggressive cancers are not missed.

The role of soluble E-cadherin (sE-cadherin) in the progression and metastasis of prostate cancer is well-documented.sE-cadherin, a cleaved form of membrane-bound E-cadherin, disrupts cellular adhesion and promotes epithelial-mesenchymal transition (EMT), a key step in cancer metastasis (27, 50). Elevated levels of sE-cadherin in the bloodstream have been associated with increased tumor invasiveness and poorer patient outcomes, making it a valuable marker for assessing the metastatic potential of prostate cancer (36, 51, 52)​.

In this study, higher sE-cadherin levels were observed in patients with advanced-stage prostate cancer (III-IV), as well as in those with bone metastasis and higher Gleason scores. This finding underscores the role of sE-cadherin as a marker of tumor progression and aggressiveness. Previous research has suggested that sE-cadherin can serve not only as a diagnostic marker but also as a potential therapeutic target (29, 40, 53). By blocking the cleavage or activity of sE-cadherin, it may be possible to inhibit the EMT process and slow the progression of metastatic prostate cancer.The integration of sE-cadherin with PHI is an innovative step that could enhance the current prostate cancer diagnostic paradigm. Comparatively, other emerging diagnostic technologies, such as multiparametric MRI (mpMRI) and next-generation sequencing (NGS), are also showing promise in improving early detection and risk stratification (54). While mpMRI has improved the detection of clinically significant prostate cancers and reduced unnecessary biopsies, its accessibility and cost can be limiting factors in some healthcare settings.Biomarker-based diagnostics, such as the combination of PHI and sE-cadherin, may offer a more accessible and cost-effective alternative, especially when used as a preliminary screening tool before more invasive or expensive procedures like MRI (55).

Moreover, next-generation sequencing techniques, which provide insights into the genetic alterations driving prostate cancer, hold promise for personalizing treatment and improving outcomes. However, these techniques are not yet widely available and can be cost-prohibitive.The biomarker approach, particularly with markers like sE-cadherin and PHI that are reflective of both tumor biology and metastatic potential, could serve as a bridge between traditional PSA testing and more advanced molecular diagnostics (56). The combination of these biomarkers could also help reduce overtreatment by better identifying patients who are at higher risk for aggressive disease and thus require more intensive monitoring and treatment.

## Clinical implications and future directions

The clinical implications of these findings are significant. Combining PHI with sE-cadherin offers a non-invasive, accessible diagnostic tool that could improve early detection rates for prostate cancer while also providing valuable information about the aggressiveness of the disease.This approach could be particularly useful in clinical settings where the goal is to minimize unnecessary biopsies and treatments for low-risk patients while ensuring that high-risk individuals receive timely intervention.However, despite the promising results, there are several limitations to the study that must be addressed before these biomarkers can be widely implemented in clinical practice.First, the study’s single-center design and relatively small sample size limit the generalizability of the findings.Larger, multicenter studies are needed to validate these results and confirm the utility of combining sE-cadherin and PHI across diverse patient populations.

Additionally, the cost-effectiveness of integrating these biomarkers into routine clinical practice must be evaluated. While biomarker testing is generally less expensive than advanced imaging or genetic sequencing, the long-term economic impact of widespread biomarker screening needs further exploration. Furthermore, standardizing the use of these biomarkers in clinical guidelines will require collaboration between researchers, clinicians, and regulatory bodies to ensure that the tests are both accurate and accessible.

## Conclusion

The combination of sE-cadherin and PHI represents a significant advancement in the field of prostate cancer diagnostics.By providing a more comprehensive assessment of both tumor presence and metastatic potential, this approach has the potential to enhance early detection, reduce overdiagnosis, and improve patient outcomes.However, further research is needed to validate these findings in larger and more diverse populations, and to explore the potential for integrating these biomarkers into clinical practice alongside other emerging diagnostic technologies.If validated, this combination of biomarkers could become a cornerstone of prostate cancer screening, helping to personalize treatment and improve long-term survival rates.

## Data Availability

The datasets presented in this study can be found in online repositories. The names of the repository/repositories and accession numbers can be found in the article/supplementary material.
